# Thermal colloid programming

**DOI:** 10.1038/s41598-025-97484-4

**Published:** 2025-04-12

**Authors:** Raphael Fortulan, Noushin Raeisi Kheirabadi, Alessandro Chiolerio, Andrew Adamatzky

**Affiliations:** 1 Unconventional Computing Laboratory, UWE, Bristol, UK; 2 School of Computing and Engineering, University of Huddersfield, Huddersfield, UK; 3 Bioinspired Soft Robotics, Istituto Italiano di Tecnologia, Via Morego 30, Genova, Italy

**Keywords:** Colloidal systems, Boolean logic, Gold nanoparticles, Unconventional computing, Temperature control, Computer science, Electrical and electronic engineering, Other nanotechnology

## Abstract

This paper investigates the computational capabilities of colloidal systems, focusing on the integration of Boolean logic operations within gold nanoparticle suspensions under varying temperature conditions. As climate change, artificial intelligence, and privacy concerns present increasing challenges for massively parallel and low-power computing devices, there is a growing demand for novel computing substrates, and colloids offer a promising avenue for developing energy-efficient and locally deployable systems. Our research explores how temperature impacts the behavior of suspended nanoparticles and, consequently, their interactions and computational performance. Our findings demonstrate that colloidal systems can perform Boolean operations, which can be modulated through temperature changes. By showcasing the versatility of these systems, this study underscores the significance of exploring unconventional computing paradigms and lays the foundation for future research into liquid-based computational applications.

## Introduction

As we progress into the 21^st^ century, facing an era of dramatic climatic changes and evolving privacy/artificial intelligence challenges, alternative computing approaches are emerging to complement and potentially mitigate limitations of established models^1^. These novel approaches offer the potential for local deployment and enhanced energy efficiency, addressing critical needs in our changing technological landscape.

For example, fluid-based computing systems, which employ incompressible fluids as computational media, represent one such alternative. These encompass various technologies, including hydraulic calculators^2,3^, fluid-based integrators^4,5^, and fluidic logic devices^6,7^. Colloids, which are suspensions of nanoparticles, represent a class of fluid-based computing that has not been fully explored. These systems, characterized by particles interacting through nonspecific forces such as van der Waals attraction and electrostatic repulsion, exhibit a natural tendency towards equilibrium. However, the application of external electric fields dramatically alters the energy landscape of these polarizable particles, propelling them away from equilibrium and onto new thermodynamic configurations. This phenomenon facilitates the emergence of complex structures, representing distinct metastable states modulated by the applied field^8–10^. At the same time, colloids show an inherent potential primarily arising from the dynamic behavior of particles within the fluid medium. Notably, the particles in the colloid exhibit a thermal motion, behavior analogous to that observed in atoms due to thermal fluctuations^11^.

Nevertheless, recent research has begun to demonstrate the potential of colloids in computational applications. Pioneering studies on liquid cybernetics have suggested employing colloidal autonomous soft holonomic processors^12^. Our research group has conducted experiments that demonstrate the computational capabilities of various colloidal systems, including proteinoid colloids^13^. In these systems, we successfully implemented Boolean logic gates. However, in our previous studies, we maintained a constant temperature, which is a crucial factor influencing the electrical properties of colloids^14,15^.

Gold nanoparticles (AuNP) suspensions/colloids, historically known as *Aurum Potabile*, have been used since medieval times for their alleged medicinal properties^16,17^. In modern times, AuNP colloids continue to be relevant in biomedical applications^18,19^ and have found new uses in data storage, optical sensing, energy transport, and computing^20,21^. In this work, we extend our prior research on AuNP colloids^22^ by investigating the effects of temperature modulation on the performance of Boolean operations within the colloidal system. Our results show that temperature directly influences the Boolean functions generated by the colloidal system, with higher temperatures leading to increased complexity and sensitivity of the Boolean expressions. This temperature dependence demonstrates that the system can be effectively controlled, expanding its potential applications in unconventional computing.

## Materials and methods

### Preparation of AuNP colloids

An aqueous suspension of Au nanoparticles, 0.1  mg ml$^{-1}$ and 14 nm particle size, was supplied by PlasmaChem GmbH (Germany). The suspension was subjected to ultrasonic treatment (using a DK Sonic Ultrasonic cleaner, UK, operating at 40  kHz) for a duration of 20 min.

### Boolean expression extraction

The extraction of 4-bit Boolean expressions from colloids was performed by applying binary strings $x \in \lbrace 0,1\rbrace ^n$, where *n* = 4. We note that the cardinality of $\vert \lbrace 0,1\rbrace \vert ^n=2^n$, thus 16 strings were used.

These strings were submitted to the colloid, with each bit changing every 30 s. The signals were encoded using alternating positive-negative polarity pulses, with logical *False* (0) represented as $-$5  V and logical *True* (1) as 5 V.

**Fig. 1:**
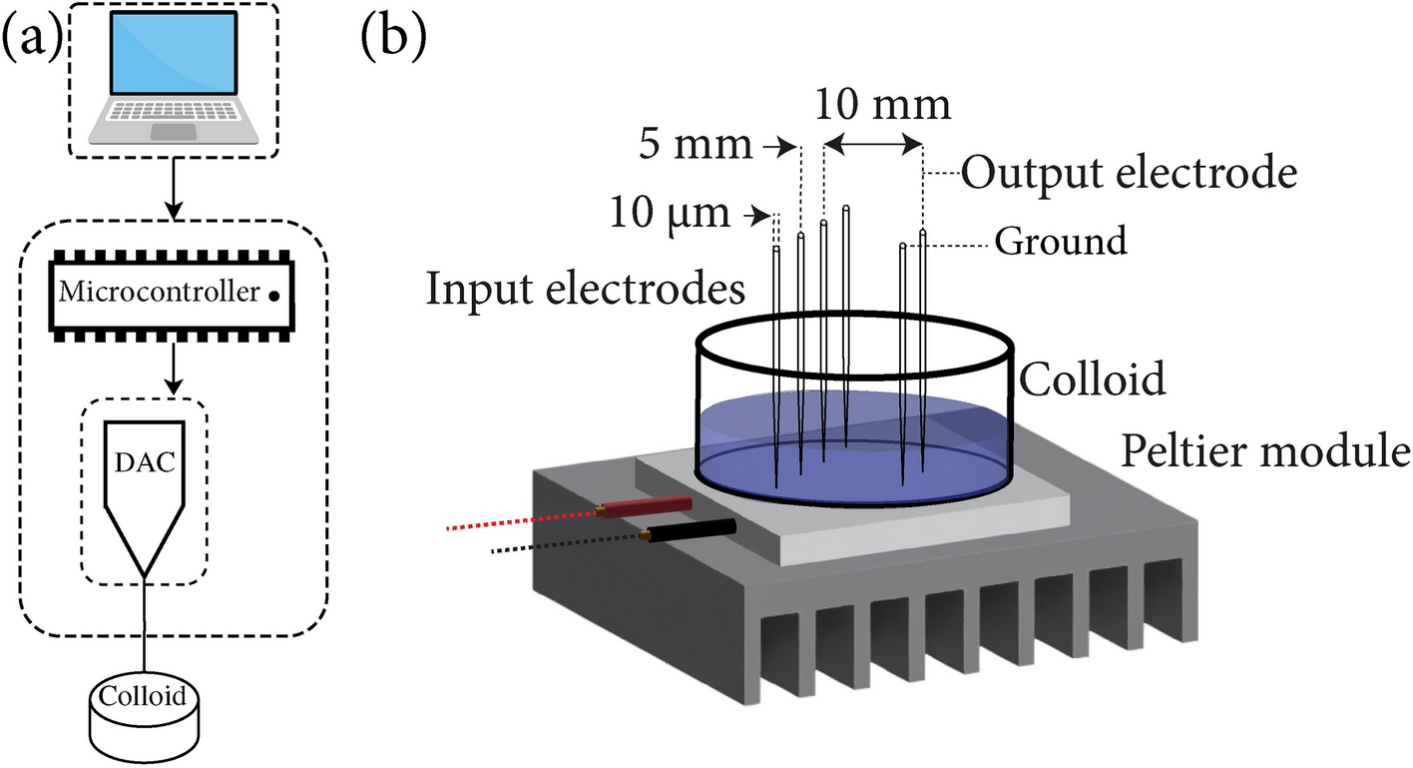
(**a**) Experimental setup for extracting Boolean expressions. (**b**) Render of the electrode arrangement for the binary string input and measured output voltages and Peltier module.

A custom-designed apparatus was employed for the extraction of the Boolean expressions, as illustrated in Fig. 1. Electrical signals were generated using an Arduino Uno R4 board (Arduino, Italy) connected via I2C to two MCP4728 12-bit quad-channel digital-to-analog converters (DACs, Microchip Technology, USA), utilizing a 74HC595 multiplexer (Texas Instruments, USA). The DAC output channels were connected to amplifier/level shift circuits based on LM258AP op-amps (Texas Instruments, USA) to produce bipolar outputs at ± 5  V. An IPS 4303 Laboratory DC Power Supply (ISO-TECH, Taiwan) provided both positive and negative voltage rails. The outputs were then electrically interfaced with the colloids using 10$\mu$m diameter platinum/iridium-coated stainless steel probes as inputs. An output probe was inserted into the colloid, and the output potential was measured using a 24-bit data logger (Pico Technology, UK), see Fig. 1a. The temperature of the colloid was regulated using a Peltier module (European Thermodynamics, 39.6 W, 12 V, UK) equipped with a fan and heatsink for enhanced heat dissipation on the hot side (Fig. 1b). Temperature control was achieved using a bang-bang controller scheme (Elitech STC-1000, UK), where the module alternated between full-on and off modes until the temperature stabilized within ± 0.3 K of the set point.

The measured output signals were subsequently threshold-classified, from 100 mV to 1 V, by assigning a logical *True* if the absolute voltage exceeded the threshold, a logical *False* if it fell below. These threshold-classified outputs were then used to create a truth table, which was subsequently converted to a Boolean expression.

### Impedance spectroscopy

Impedance spectroscopy was performed using an Inductance-Capacitance-Resistance (LCR) meter (model 891, BK Precision Ltd, UK). The meter was configured to scan across a frequency range of 20 to 300 kHz, using a 0.5 $\hbox {V}_\textrm{rms}$ sinusoidal voltage waveform.

## Results and discussion

### Boolean functions extraction

Boolean expressions characterizing the AuNP colloid have been derived at six different temperatures between almost freezing and boiling points of $\hbox {H}_{2}$O: 273.65, 278.15, 283.15, 293.15, 323.15, and 343.25 K, as detailed in Tables 1, 2, 3, 4, 5, 6. The different Boolean expressions at each temperature result from the different threshold values used at each temperature.

To assess how temperature influences the complexity of the extracted logic gates, the query complexity of all Boolean functions was evaluated. This measure quantifies the number of queries necessary to evaluate a Boolean function $f:\lbrace 0,1\rbrace ^n\rightarrow \lbrace 0,1\rbrace$. To proceed with this evaluation, we first define a decision tree as follows:

#### Definition 1

(Decision tree) A decision tree is a binary tree with each internal node labeled with one of *n* input Boolean variables. One of the two edges leaving the node is labeled 0 (or *False*), and the other is labeled 1 (or *True*). Each leaf is also labeled 0 (or *False*) or 1 (or *True*). The two sub-trees at each node describe how the algorithm will proceed after receiving an answer to the query. The two labels represent the two possible answers to the query. The label of the reached leaf is the value of the function for that particular input.

The depth of a decision tree is the longest path from root to leaf. Equivalently, we can define the following metric:

#### Definition 2

(Decision tree cost) The cost of a tree $t\in {\mathcal {T}}$ for an input *x*, $\textrm{cost}(x, t),$ is the number of queries evaluated in *t* when executing *x*.

The depth of a tree is then $\max _X \textrm{cost}(x, t)$, where *X* is the set of all possible Boolean inputs for *f*. Finally, the complexity of a Boolean function is defined as:

#### Definition 3

(Decision tree complexity) The complexity ${\mathcal {C}}$ of a function *f* depth of the minimal decision tree, or the most efficient, that computes *f*

1 $$\begin{aligned} {\mathcal {C}}(f)=\min _{t\in {\mathcal {T}}}\max _{x\in X}\textrm{cost}(x, t). \end{aligned}$$

Note that since evaluating $f:\lbrace 0,1\rbrace ^n\rightarrow \lbrace 0,1\rbrace$ *n* times fully compute *f*, ${\mathcal {C}}(f)\leqslant n$.

The depth of the most efficient decision tree that computes is not the only way of gauging the complexity of a Boolean expression. Cook, Dwork, and Reischuk^23,24^ originally introduced sensitivity, also known as critical complexity, as a simple combinatorial complexity measure for Boolean functions. Intuitively, as the name suggests, the sensitivity of a function measures the number of bits of the input on which the function is sensitive. The formal definition is given below.

#### Definition 4

(Sensitivity of a Boolean function) The sensitivity, *s*(*f*) of a Boolean function $f:\lbrace 0,1\rbrace ^n\rightarrow \lbrace 0,1\rbrace$ at *x* is defined as $s = \vert \lbrace i: f(x) \ne f(x\oplus \mathbbm {1}_i\rbrace \vert$, where $\oplus$ is the XOR operation and $\mathbbm {1}_i$ is a Boolean vector of length *n* with all 0s except a the *i*-th position.

#### Definition 5

(Average sensitivity of a Boolean function) The average sensitivity, ${\bar{s}}(f)$ of a Boolean function $f:\lbrace 0,1\rbrace ^n\rightarrow \lbrace 0,1\rbrace$ across all possible inputs *x* is

2 $$\begin{aligned} {\bar{s}}(f) = \frac{\sum _x s(f)}{2^{n}}. \end{aligned}$$

#### Definition 6

(Block sensitivity of a Boolean function) The block sensitivity, *bs*(*f*) of a Boolean function $f:\lbrace 0,1\rbrace ^n\rightarrow \lbrace 0,1\rbrace$ at *x* is defined as the maximum number of disjoint subsets of $B^{j}_{lk}\in \lbrace 1,2,\dots ,n \rbrace$ such that $\forall j, f(x) \ne f(x \oplus \mathbbm {1}_{B^{j}_{lk}})$, where $\mathbbm {1}_{B^{j}_{lk}}$ is the Boolean vector of size *n* with all 0s except in the positions assigned by ${B^{j}_{lk}}$.

#### Definition 7

(Average block sensitivity of a Boolean function) The average block sensitivity, ${\bar{bs}}(f)$ of a Boolean function $f:\lbrace 0,1\rbrace ^n\rightarrow \lbrace 0,1\rbrace$ across all possible inputs *x* is

3 $$\begin{aligned} {\bar{bs}}(f) = \frac{\sum _x bs_{x}(f)}{2^{n}}. \end{aligned}$$

**Table 1:** Extracted Boolean expression at 273.65 K, their complexity ${\mathcal {C}}$, average sensitivity ${\bar{s}}$, and average block sensitivity ${\bar{bs}}$.

Boolean expression	${\mathcal {C}}$	${\bar{s}}$	${\bar{bs}}$
*True*	0	0	0
*False*	0	0	0
B $\vee$ C $\vee$ $\lnot$D	3	0.75	2.87
C $\vee$ (A $\wedge$ $\lnot$D) $\vee$ (B $\wedge$ $\lnot$D)	4	1.25	3.62
C $\vee$ (A $\wedge$ $\lnot$B $\wedge$ $\lnot$D) $\vee$ (B $\wedge$ $\lnot$A $\wedge$ $\lnot$D)	4	1.5	3.75
(B $\wedge$ C) $\vee$ (A $\wedge$ $\lnot$B $\wedge$ $\lnot$C $\wedge$ $\lnot$D)	4	1.5	3.37

**Table 2:** Extracted Boolean expression at 278.15 K, their complexity ${\mathcal {C}}$, average sensitivity ${\bar{s}}$, and average block sensitivity ${\bar{bs}}$.

Boolean expression	${\mathcal {C}}$	${\bar{s}}$	${\bar{bs}}$
*True*	0	0	0
A $\vee$ C	2	1	3.5
(A $\wedge$ B) $\vee$ (A $\wedge$ D) $\vee$ (B $\wedge$ C) $\vee$ (C $\wedge$ $\lnot$D)	4	1.5	3.75
(A $\wedge$ C) $\vee$ (B $\wedge$ C) $\vee$ (C $\wedge$ $\lnot$ D)	4	1.25	3.87
(A $\wedge$ B $\wedge$ C) $\vee$ (A $\wedge$ C $\wedge$ $\lnot$D)	4	1	3.18
A $\wedge$ B $\wedge$ C	3	0.75	2.87
*False*	0	0	0

**Table 3:** Extracted Boolean expression at 283.15 K, their complexity ${\mathcal {C}}$, average sensitivity ${\bar{s}}$, and average block sensitivity ${\bar{bs}}.$.

Boolean expression	${\mathcal {C}}$	${\bar{s}}$	${\bar{bs}}$
*True*	0	0	0
B $\vee$ C $\vee$ (A $\wedge$ $\lnot$D)	4	1	3.18
C $\vee$ (A $\wedge$ $\lnot$D) $\vee$ (B $\wedge$ $\lnot$D)	4	1.25	3.62
C $\vee$ (A $\wedge$ $\lnot$D)	3	1.25	3.75
(B $\wedge$ C) $\vee$ (A $\wedge$ $\lnot$B $\wedge$ $\lnot$C $\wedge$ $\lnot$D)	4	1.5	3.37
B $\wedge$ C $\wedge$ $\lnot$A $\wedge$ $\lnot$D	4	0.5	2.25
*False*	0	0	0

**Table 4:** Extracted Boolean expression at 293.15 K, their complexity ${\mathcal {C}}$, average sensitivity ${\bar{s}}$, and average block sensitivity ${\bar{bs}}$.

Boolean expression	${\mathcal {C}}$	${\bar{s}}$	${\bar{bs}}$
*True*	0	0	0
A $\vee$ $\lnot$C $\vee$ $\lnot$D	3	0.5	2.25
(A $\wedge$ B) $\vee$ (A $\wedge$ $\lnot$C) $\vee$ (B $\wedge$ $\lnot$C) $\vee$ (C $\wedge$ $\lnot$D) $\vee$ (D $\wedge$ $\lnot$C)	4	1	3.18
(A $\wedge$ $\lnot$C) $\vee$ (D $\wedge$ $\lnot$C) $\vee$ (A $\wedge$ B $\wedge$ D) $\vee$ (A $\wedge$ $\lnot$B $\wedge$ $\lnot$D)	4	1.25	3.75
(A $\wedge$ $\lnot$C) $\vee$ (A $\wedge$ B $\wedge$ D) $\vee$ (D $\wedge$ $\lnot$B $\wedge$ $\lnot$C)	4	1.5	3.75

**Table 5:** Extracted Boolean expression at 323.15 K, their complexity ${\mathcal {C}}$, average sensitivity ${\bar{s}}$, and average block sensitivity ${\bar{bs}}$.

Boolean circuit	${\mathcal {C}}$	${\bar{s}}$	${\bar{bs}}$
*False*	0	0	0
C $\vee$ D $\vee$ $\lnot$A $\vee$ $\lnot$B	4	0.5	2.25
$\lnot$A $\vee$ $\lnot$B	2	1	3.5
($\lnot$B $\wedge$ $\lnot$C) $\vee$ (A $\wedge$ $\lnot$B $\wedge$ $\lnot$D) $\vee$ (B $\wedge$ C $\wedge$ D $\wedge$ $\lnot$A)	4	1.75	3.5
(D $\wedge$ $\lnot$B $\wedge$ $\lnot$C) $\vee$ ($\lnot$A $\wedge$ $\lnot$B $\wedge$ $\lnot$C) $\vee$ (A $\wedge$ C $\wedge$ $\lnot$B $\wedge$ $\lnot$D)	4	1.5	3.37
(A $\wedge$ C $\wedge$ $\lnot$B $\wedge$ $\lnot$D) $\vee$ (A $\wedge$ D $\wedge$ $\lnot$B $\wedge$ $\lnot$C)	4	1	2.75

**Table 6:** Extracted Boolean expression at 343.15 K, their complexity ${\mathcal {C}}$, average sensitivity ${\bar{s}}$, and average block sensitivity ${\bar{bs}}$.

Boolean expression	${\mathcal {C}}$	${\bar{s}}$	${\bar{bs}}$
(A $\wedge$ $\lnot$B) $\vee$ (B $\wedge$ $\lnot$A) $\vee$ (B $\wedge$ $\lnot$C $\wedge$ $\lnot$D)	4	2	3.12
$\lnot$D $\vee$ (B $\wedge$ C) $\vee$ (A $\wedge$ $\lnot$B) $\vee$ ($\lnot$A $\wedge$ $\lnot$C)	4	1	2.87
(B $\wedge$ C) $\vee$ (A $\wedge$ $\lnot$B) $\vee$ ($\lnot$A $\wedge$ $\lnot$C) $\vee$ ($\lnot$C $\wedge$ $\lnot$D)	4	1.25	2.93
(B $\wedge$ C) $\vee$ (A $\wedge$ $\lnot$B) $\vee$ (B $\wedge$ $\lnot$A) $\vee$ (B $\wedge$ $\lnot$D)	4	1.5	3.37
(A $\wedge$ $\lnot$B) $\vee$ (B $\wedge$ $\lnot$A) $\vee$ (B $\wedge$ C $\wedge$ D) $\vee$ (B $\wedge$ $\lnot$C $\wedge$ $\lnot$D)	4	2	3.25
(A $\wedge$ C $\wedge$ $\lnot$B) $\vee$ (A $\wedge$ D $\wedge$ $\lnot$B) $\vee$ (B $\wedge$ C $\wedge$ $\lnot$A) $\vee$ (B $\wedge$ D $\wedge$ $\lnot$A) $\vee$ (A $\wedge$ B $\wedge$ $\lnot$C $\wedge$ $\lnot$D)	4	2.5	3.31

**Fig. 2:**
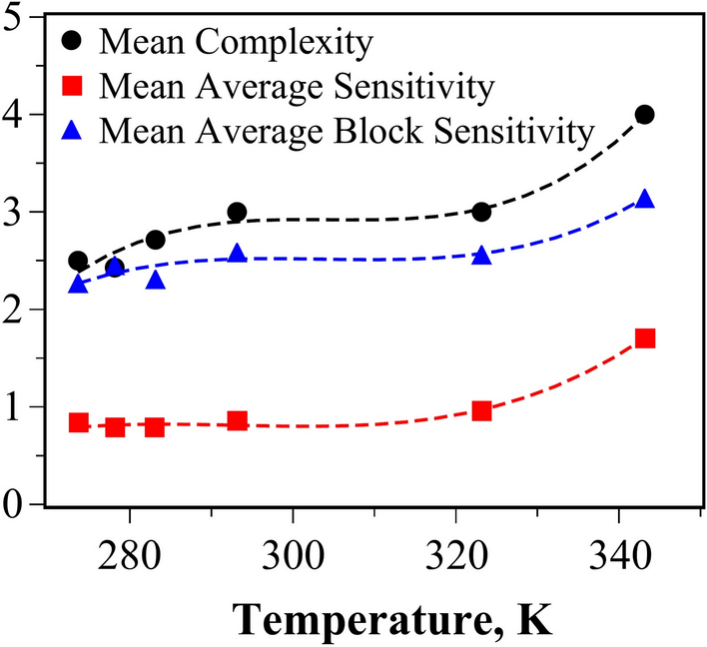
Evolution of the mean value for complexity, average sensitivity, and average block sensitivity as the temperature of the colloid increases. The dashed lines represent a polynomial interpolation of the values, serving as a visual aid to illustrate the overall increase.

The calculated values for query complexity, average sensitivity, and average block sensitivity of the Boolean expressions across all temperature ranges are presented in Tables 1, 2, 3, 4, 5, 6. Analysis of these Boolean expressions across different temperatures (from 273.65 K to 343.15 K) reveals several key trends. The complexity of the expressions remains relatively consistent, predominantly ranging between 3 and 4 terms, with minimal variation across temperature changes. Both average sensitivity and average block sensitivity exhibit a slight increase at elevated temperatures, particularly at 323.15 K and 343.15 K. This suggests that as temperature rises, the Boolean expressions become more susceptible to changes in their input variables, indicating that small fluctuations in input are more likely to alter the outcome. Block sensitivity, which reflects the impact of changes to groups of variables, remains relatively stable across all temperatures, typically ranging from 2.25 to 3.87. The overall pattern indicates that temperature influences the system’s responsiveness, with higher temperatures corresponding to increased sensitivity. In Fig. 2, the mean values for complexity, average sensitivity, and average block sensitivity at each temperature are plotted against the temperature of the colloid. The dashed line, representing a polynomial interpolation of the data, highlights the increase in values as the temperature rises.

**Fig. 3:**
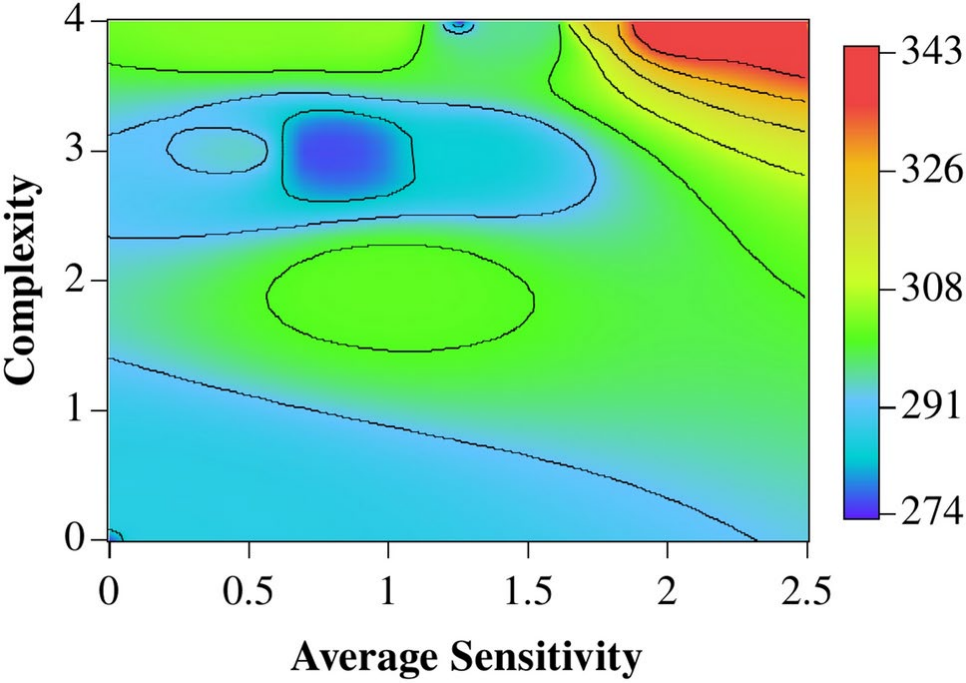
Interpolated heatmap of calculated complexity and average sensitivity of the Boolean expressions extracted for all temperature range. The contour lines were added to indicate regions with same temperature.

Graphically, the relationship between query complexity, average sensitivity, and temperature is clearly illustrated in Fig. 3, where regions of higher temperatures correspond to higher values for both variables. If on the one hand it is clear that the higher sensitivities cannot be found at the lowest temperatures, the higher complexities seem to be less influenced by temperatures.

The enhanced sensitivity at higher temperatures suggests potential applications of thermally-activated colloidal systems in Boolean-based cryptographic applications^25,26^. In this context, highly sensitive Boolean functions can enhance the resilience of cryptographic algorithms against attacks such as differential and linear cryptanalysis. Furthermore, sensitive functions can mitigate the risk of side-channel attacks, where adversaries attempt to extract information from the physical implementation of the cryptographic algorithm^27^.

**Fig. 4:**
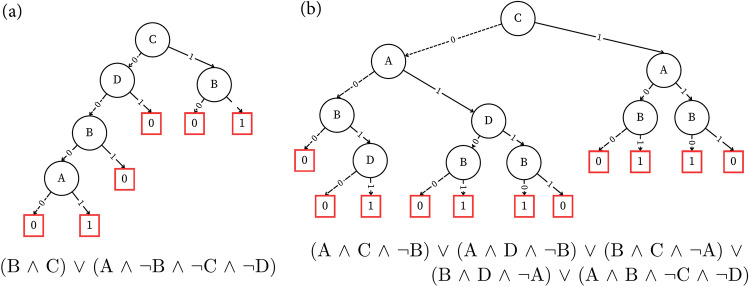
Minimal decision tree for Boolean functions extracted at (**a**) 273.65 K (**b**) 343.15 K.

This trend can also be visualized by comparing the decision trees of Boolean functions extracted at near-freezing temperature and at 343.15 K, as seen in Fig. 4. It’s evident that although the depth of the trees remains constant, the increased number of nodes and spatial complexity lead to a higher sensitivity in the Boolean function at higher temperatures.

### Mechanisms for computational capability

The computational capabilities of the colloid may arise from two primary mechanisms. First, given the particle size of 14 nm and a gold density of 19.3 g cm$^{-3}$^28^, the estimated number of particles per ml is approximately 3.59 $\times 10^{12}$ particles $\hbox {ml}^{-1}$, with an average center-to-center distance of 653 nm. At this distance, the electromagnetic field enhancement is observable, as indicated by the colloidal deep purple color. Consequently, there is an interaction mediated by light, which is further enhanced by surface plasmons-collective oscillations of electrons on the particle surfaces^29^. This plasmonic effect has already been exploited for computational purposes^30^.

Simultaneously, we observe the Brownian motion of particles^31,32^, electrostatic repulsive interactions due to overlapping electrical double layers surrounding the particles, and van der Waals attractive interactions between particles^33^. The Brownian motion, in particular, indicates that the collision rate and kinetic energy of the particles increase with temperature. Generally, this results in faster aggregation^34^ and changes the electrical properties of the suspension.

**Fig. 5:**
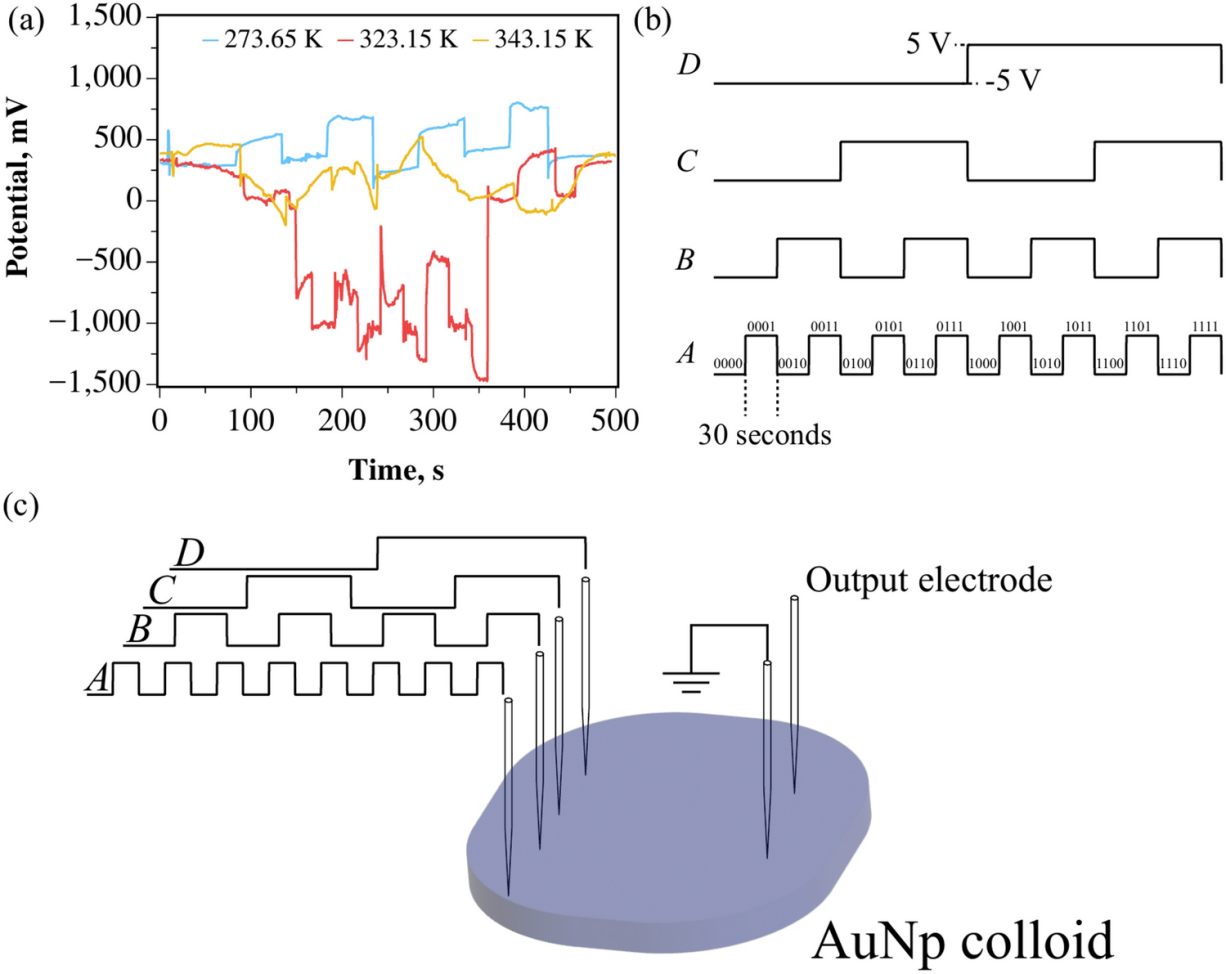
(**a**) Measured output potential at 273.65 K, 323.15 K, and 343.25 K. (**b**) Diagram and corresponding Boolean strings for four inputs into the colloid. (**c**) Illustration of the applied Boolean strings into the AuNp colloid.

In Fig. 5a, the temperature-dependent modulation in the recorded output potential of the colloid for binary string inputs, as shown in Fig. 5b,c, can be clearly seen at temperatures of 273.65, 323.15, and 343.25 K.

At lower temperatures, such as 273.65 K, the system exhibits a linear response to the inputs. As the temperature increases, the system’s response becomes progressively more nonlinear. This change is likely due to the increased thermal energy of the colloidal particles, which augments their Brownian motion and interaction frequency^9,11,35^. The more frequent and complex interactions at elevated temperatures resulted in a marked increase in the complexity measures of the Boolean expressions derived from the system.

**Fig. 6:**
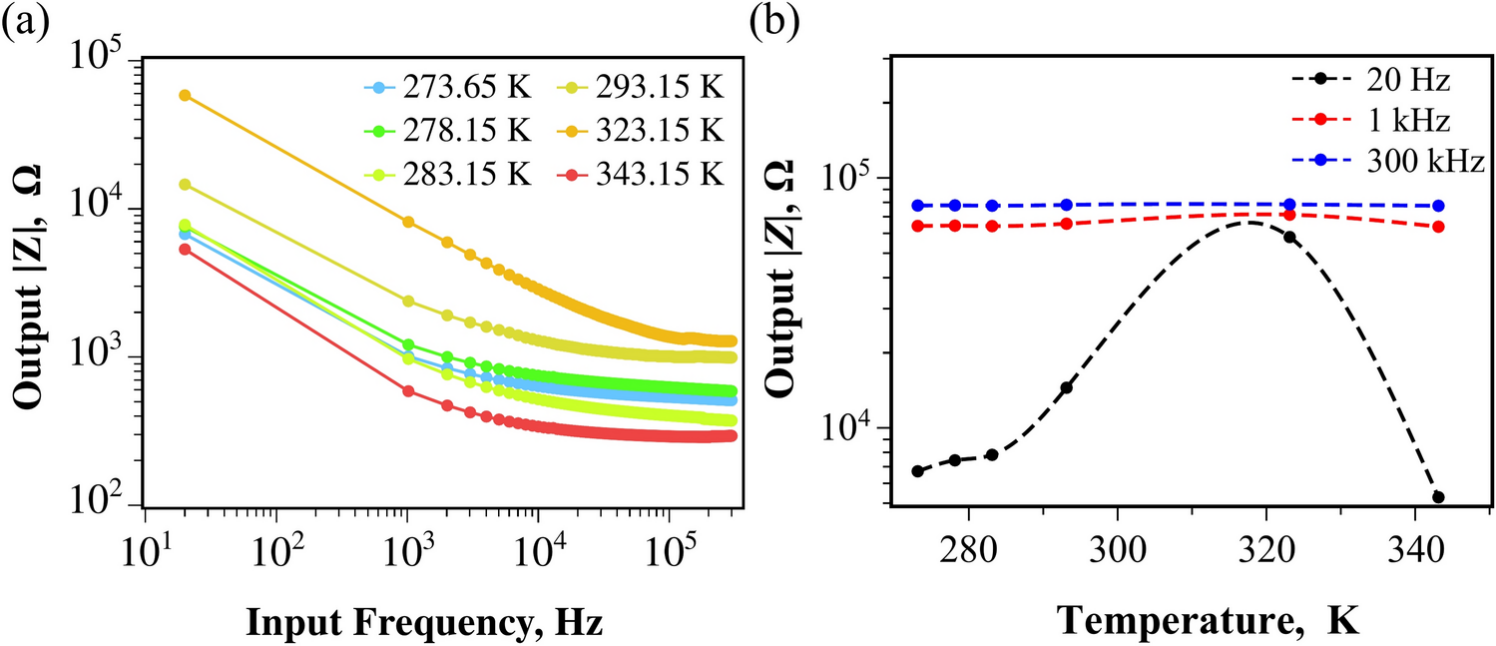
(**a**) Impedance spectroscopy of the AuNP colloid at 273.65, 278.15, 283.15, 293.15, 323.15, and 343.25 K. (**b**) Temperature dependence of the impedance of the AuNP colloid at input frequencies of 20, 1 , and 300 kHz.

The changes observed in the AuNP colloid can be further characterized through impedance spectroscopy analysis of the suspension at various temperatures. As shown in Fig. 6a, temperature modulates the complex impedance (*Z*) of the colloidal system, with an increase observed up to 323.15 K, followed by a decrease at 343.15 K.

The increase in impedance as the temperature rises to 323.15 K indicates a decrease in the electrical conductivity of the samples similar to what has been observed in theoretical and experimental analysis of thermal conductivity of colloids with very small nanoparticles^36,37^.

The subsequent decrease in impedance at 343.15 K suggests a structural change in the colloidal system. It is well established that nanoparticle aggregation depends on both time and temperature^38^, with higher temperatures promoting this effect. At the highest measured temperature, the aggregated particles likely formed structures that resonated with each other at higher signal frequencies (above 10 kHz), enhancing charge transport and reducing electrical impedance.

We note that the changes in impedance and temperature are not linear, which aligns with the nonlinear dependence of particle size on temperature in colloids^39,40^. Additionally, the relationship between particle size and impedance is also nonlinear, which alters $\vert Z\vert$ as the input frequency changes^40,41^ as seen in Fig. 6b.

In previous reports^42^, it was observed that colloids exhibit memfractance properties, meaning the system demonstrates an interpolated behavior between a memristor, a second-order memristor, and a memcapacitor^43^. These properties introduce a high level of nonlinearity between the voltage applied to the material, resulting in significant complexity in the extracted Boolean functions. Furthermore, we observed that by varying the temperature of the system, we achieved higher complexity values compared to room-temperature experiments. As indicated by changes in the system’s impedance, this behavior is likely due to the temperature dependence of the material’s capacitive and resistive properties

## Potential applications

The possibility of controlling the Boolean logic extracted from the colloids supports the idea that the colloids can be used for more sophisticated operations. As an illustration, we show how one of the extracted Boolean functions can be mapped to a 4:1 multiplexer (MUX).

For example, consider the function (A$\wedge \lnot$C)$\vee$(D$\wedge \lnot$C)$\vee$(A$\wedge$B$\wedge$D)$\vee$(A$\wedge \lnot$B$\wedge \lnot$D). Let us assume four data inputs, $\hbox {I}_0$, $\hbox {I}_1$, $\hbox {I}_2$, and $\hbox {I}_3$ and two select lines, $\hbox {S}_0$ and $\hbox {S}_1$. We can define the following mapping

1. $\hbox {I}_0$ corresponds to (A$\wedge \lnot$C)$\vee$(D$\wedge \lnot$C) when $\hbox {S}_0$ = 1 and $\hbox {S}_1$ = 0,
2. $\hbox {I}_1$ corresponds to (A$\wedge$B$\wedge$D) when $\hbox {S}_0$ = 1 and $\hbox {S}_1$ = 0,
3. $\hbox {I}_2$ corresponds to (A$\wedge \lnot$B$\wedge \lnot$D) when $\hbox {S}_0$ = 0 and $\hbox {S}_1$ = 0,
4. $\hbox {I}_3$ corresponds to 0 when $\hbox {S}_0$ = 1 and $\hbox {S}_1$ = 1.

Based on this mapping, we can define the following MUX expression

4 $$\begin{aligned} {Y=(I_0\wedge \lnot S_1\wedge \lnot S_0)\vee (I_1\wedge \lnot \wedge S_0)\vee (I_2\wedge S_1\wedge \lnot S_0)\vee (I_3\wedge S_1\wedge S_0)}. \end{aligned}$$

This mapping to a 4:1 multiplexer demonstrates that the Boolean functions emerging from our temperature-controlled colloidal system are not just abstract logical operations, but can be interpreted as building blocks for practical computational devices. This suggests that colloidal systems could potentially be used to create temperature-sensitive logic circuits or reconfigurable computing elements.

## Conclusions

Our study demonstrates the significant potential of AuNP colloids as a platform for temperature-controlled Boolean logic operations, marking a substantial advancement in unconventional computing. We have shown that these colloids can effectively perform Boolean logic operations, with their capabilities modulated by temperature changes. As temperature increases, the complexity and sensitivity of the extracted Boolean functions tend to rise, suggesting enhanced computational potential at elevated temperatures.

This temperature-dependent modulation is likely due to increased Brownian motion and enhanced interactions between nanoparticles. Our analysis revealed also nonlinear relationship between temperature and the electrical properties of the colloidal system, correlating with the observed changes in computational behavior.

These findings open up new avenues for developing temperature-sensitive and reconfigurable computing elements . Potential applications span various fields, including cryptography, biomedical engineering, and environmental monitoring. Future research should focus on expanding the temperature range, exploring other environmental factors influencing colloidal computing, and investigating the scalability and stability of these systems for practical applications.

## Data Availability

Data sets generated during the current study are available from at 10.5281/zenodo.15200796.
